# METTL3‐induced FGD5‐AS1 contributes to the tumorigenesis and PD‐1/PD‐L1 checkpoint to enhance the resistance to paclitaxel of endometrial carcinoma

**DOI:** 10.1111/jcmm.17971

**Published:** 2023-09-27

**Authors:** Min Hao, Tianjie Li, Ling Xiao, Yun Liu

**Affiliations:** ^1^ Department of Obstetrics and Gynecology Beijing Friendship Hospital Affiliated to Capital Medical University Beijing China

**Keywords:** endometrial carcinoma, FGD5‐AS1, immune checkpoint, METTL3, paclitaxel

## Abstract

Endometrial cancer (EC), a widely occurring cancer in the uterus, is among the top four most frequent malignancies in women. To improve approaches for combating this disease, it is essential to gain a more comprehensive comprehension of the intricate causes of EC. Accumulating evidence highlight the essential role of long non‐coding RNA (LncRNA) in EC progression, while its biological and mechanical function has not been fully revealed. In this study, a LncRNA microarray analysis was performed using four pairs of paclitaxel (PTX) resistant EC cells, FGD5‐AS1 was identified as a significantly upregulated gene. Biologically, it was found that FGD5‐AS1 enhances chemoresistance of EC cells to PTX treatment and blocking immune escape via PD‐1/PD‐L1 checkpoint. Furthermore, FGD5‐AS1 exerted an oncogene role in EC cells via promoting cell proliferation and migration. Mechanically, METTL3 could upregulate FGD5‐AS1 expression via N6‐methyladenosine (m6A) modification. The biological roles of METTL3 were exerted via modulating FGD5‐AS1 expression in EC. Collectively, our research has shed light on the involvement of the METTL3/FGD5‐AS1 axis in the development of PTX resistance in EC. This finding offers a new avenue for further exploration of the underlying mechanisms of chemoresistance in EC and provides valuable insights for the development of potential therapeutic targets in the treatment of EC.

## 1 INTRODUCTION

Among women, endometrial cancer (EC) is a highly prevalent type of cancer originating in the uterus, making it the fourth most frequently occurring malignancy.^1^ The development of mutations in the endometrium leads to tumorigenesis. EC generally has a favourable prognosis, boasting a 5‐year overall survival (OS) rate ranging from 74% to 95%. However, for patients with advanced or metastatic EC, the prognosis remains grim, with a 5‐year OS rate plummeting to a mere 15% to 17%. This grim outlook is primarily attributed to the challenges posed by tumour metastasis and recurrence in these advanced stages of the disease.^2^ Various approaches have been developed to combat EC, such as surgery, radiotherapy, chemotherapy, targeted drug therapy and immunotherapy.^3^, ^4^ However, a deeper understanding of the detailed aetiology of EC is still needed in order to enhance strategies for combating this disease.

Long non‐coding RNA (LncRNA) is a group of RNA molecules characterized by their length, typically exceeding 200 nucleotides. While the most LncRNAs do not encode proteins, some may still possess the ability to code for proteins.^5^ Numerous studies have provided substantial evidence highlighting the essential roles of LncRNAs in interacting with various cellular components, such as miRNA, mRNA, DNA and proteins. These interactions give rise to complex networks that play pivotal roles in regulating diverse biological functions, including cellular energy metabolism, susceptibility to microbial agents and epigenetic processes.^6^, ^7^ The regulatory mechanisms of LncRNA in EC have been extensively studied and documented. One notable example is the LncRNA LINC01224, which has been found to regulate the expression of AKT3 by serving as a target for miR‐485‐5p. This regulatory interaction promotes cell proliferation and simultaneously inhibits apoptosis in EC.^8^ Furthermore, the LncRNA EIF1AX‐AS1 has been identified as a key player in the progression of EC. It exerts its influence by affecting the stability of EIF1AX mRNA. Specifically, EIF1AX‐AS1 functions to promote apoptosis in EC cells and acts as an inhibitor of EC progression.^9^ Accumulating evidence highlight the essential role of LncRNA in EC progression.

In this study, FGD5‐AS1 was identified as a most overexpressed LncRNA in paclitaxel (PTX) resistant EC cells. Subsequently, the biological and molecular functions of FGD5‐AS1 in EC were investigated. Biologically, FGD5‐AS1 knockdown re‐sensitive EC cells to PTX treatment and blocking EC cell immune escape through PD‐1/PD‐L1 checkpoint. Moreover, FGD5‐AS1 knockdown attenuated EC tumorigenic properties, by inhibiting cell proliferation and migration. Mechanically, FGD5‐AS1 was found to be a downstream target of METTL3, which could regulate the N6‐methyladenosine (m6A) modification in FGD5‐AS1. Furthermore, METTL3 enhanced PTX resistance and tumorigenesis via modulating FGD5‐AS1 expression. Collectively, our results indicate that METTL3 and FGD5‐AS1 may be potential therapeutic targets for EC.

## 2 MATERIALS AND METHODS

### 2.1 Cell culture and establishment of drug‐resistant cell lines

Ishikawa and HHUA human EC cell lines were procured from the BeNa Culture Collection, Beijing, China. The cell lines were cultured following the instructions provided by the manufacturer. In the initial step, Ishikawa and HHUA cells in the logarithmic growth phase were exposed to a concentration of 0.1 μM PTX for a duration of 24 h. Following this, the cells were washed with PBS to remove the fresh medium containing PTX, and they were subsequently cultured continuously. After three passages, the concentration of PTX was gradually increased from 0.2 μM to 0.5 μM, 1.0 μM and finally to 2.0 μM, until the cells could sustain stable growth at a concentration of 2.0 μM PTX. This established a drug‐resistant cell strain. Subsequently, Ishikawa, Ishikawa‐RE, HHUA‐RE and HHUA cells were treated with the varying concentrations of PTX (0, 0.25, 0.5, 1.0, 2.5, 5.0 and 10.0 μM) to confirm the successful construction of the drug‐resistant cell lines. All cells were cultured at a temperature of 37°C with 5% CO_2_ and were collected during the logarithmic growth phase.

### 2.2 Cell transfection

Gene Pharma synthesized the plasmids for overexpressing FGD5‐AS1, which were subsequently ligated into the pAd‐Flag vector. The specific sequence of the FGD5‐AS1‐overexpressing plasmid was obtained using the following primer: 5'‐CCTGACCTTTCGCCAACTGACT‐3'. For the negative control, plasmids without any target sequence were utilized, and the corresponding primer sequence was 5'‐GATTACAAGGACGACGATGACAAG‐3'. The specific small interfering RNA (siRNA) sequences targeting FGD5‐AS1, including si‐FGD5‐AS1 #1 and si‐FGD5‐AS1 #2, were synthesized by GenePharma. The sequences for si‐FGD5‐AS1 #1 and si‐FGD5‐AS1 #2 are as follows: si‐FGD5‐AS1 #1: 5'‐CAUUUGUAAUAGUGUUCAAUA‐3', si‐FGD5‐AS1 #2: 5'‐GCGUAGUUACAAUGAUUUAAA‐3'. siRNA targeting methyltransferase‐like 14 (METTL14) (si‐METTL14, 5'‐CCGACAGCATTGGTGCCGTGTTAAA‐3') and METTL3 (si‐METTL3, 5'‐GCACTTGGATCTACGGAAT‐3') was synthesized by RiboBio. In addition, a scrambled negative control siRNA (si‐NC) was synthesized by GenePharma for use as a control in the experiments. Transfection experiments were performed using Lipofectamine 3000 transfection reagent (Invitrogen).

### 2.3 LncRNA microarray analysis

Total RNA was isolated from cultured cells using the Trizol reagent (TaKaRa) as per the manufacturer's instructions. The Affymetrix Gene ChipR Human Transcriptome Array 2.0 (Santa Clara) was used for microarray analysis, following the manufacturer's protocols.

### 2.4 Quantitative real‐time PCR analysis (qRT‐PCR)

Trizol reagent (Invitrogen) was utilized for the extraction of total RNA from EC cell lines. In addition, NE‐PER Nuclear and Cytoplasmic Extraction Reagents (Thermo Scientific) were employed to isolate RNA from the cytoplasmic and nuclear fractions of EC cells. A reverse transcriptase cDNA synthesis kit (Toyobo) was used to perform reverse transcription of the extracted RNA into cDNA. GAPDH was used as the endogenous control. Quantitative PCR (qPCR) was subsequently conducted using the SYBR Green PCR Kit (Roche) to detect RNA expression levels. The primers utilized in this study are provided below; FGD5‐AS1; Forward: 5′‐GCACGTAAAAGGCCTTTGCA‐3′, Reverse: 5′‐GCTAACGCACACACACAAGG‐3′, METTL3; Forward: 5′‐AGATGGGGTAGAAAGCCTCCT‐3′, Reverse: 5′‐TGGTCAGCATAGGTTACAAGAGT‐3′, METTL14; Forward: 5′‐GAACACAGAGCTTAAATCCCCA‐3′, Reverse: 5′‐TGTCAGCTAAACCTACATCCCTG‐3′, GAPDH; Forward: 5′‐GGAGCGAGATCCCTCCAAAAT‐3′, Reverse: 5′‐GGCTGTTGTCATACTTCTCATGG‐3′.

### 2.5 Western blot

The cells were initially washed with PBS and then subjected to lysis using RIPA buffer (Thermo Scientific). The resulting lysates were sonicated on ice and subsequently centrifuged at 14,000 *g* for 5 min at 4°C in order to remove cellular debris. The supernatants obtained were then separated on 12.5% SDS–PAGE gels (30 μg of protein per lane) and transferred onto polyvinylidene fluoride (PVDF) membranes with a pore size of 0.45 μm (Millipore). Subsequently, the membranes were incubated overnight at 4°C with primary antibodies obtained from Cell Signaling Technology, specifically rabbit anti‐METTL3 (#86132), rabbit anti‐PD‐L1 (13684) and rabbit anti‐GAPDH (D6H11). Following three washes, the membranes were incubated with secondary antibodies labelled with HRP for 2 h at room temperature. Finally, protein bands were visualized using the enhanced chemiluminescence (ECL) method.

### 2.6 Detection of CD8 ^+^ T‐cell percentage and apoptosis

Transfections were carried out on PTX‐resistant Ishikawa or HHUA cells using different constructs, while untransfected cells served as the control group. CD8^+^ T cells were isolated from healthy human peripheral blood mononuclear cells. The transfected PTX‐resistant Ishikawa or HHUA cells were then co‐cultured with CD8^+^ T cells for a duration of 12 h. Thalidomide, anti‐PD‐1, or anti‐PD‐L1 antibodies were added as indicated in the experiment. Single‐cell suspensions were prepared from the infiltrating CD8^+^ T cells and stained with a fluorescent CD8 antibody to analyse the percentage of CD8^+^ T cells. To assess apoptosis, the single‐cell suspensions were stained with an Annexin V‐FITC Apoptosis Detection Kit (ThermoFisher Scientific). Flow cytometry analysis was performed using a BD FACSCalibur instrument to measure the percentage of CD8^+^ T cells and apoptosis.

### 2.7 MTT assay

For cell proliferation analysis, cells were seeded in 96‐well plates at a density of 1 × 105 cells per well in DMEM medium supplemented with 10% FBS. At different time intervals, 20 μL of MTT dye was introduced to each well. Following incubation, the formed formazan crystals were solubilized by adding 150 μL of DMSO and incubating for 5 min. The absorbance of the resulting colour reaction was measured at 570 nm using an Enzyme Immunoassay Analyzer (Bio‐Rad, Hercules, CA). Based on the measured absorbance values, the proliferation activity was then calculated for each clone.

### 2.8 CCK‐8 assay

Cell viability was evaluated by adding 10 μL of CCK‐8 solution to each well of the plate at 24‐h intervals. The plate was then incubated at 37°C for 2 h. After the incubation, the spectrophotometric absorbance of each sample was measured at 450 nm to assess cell viability.

### 2.9 Cell colony assay

A cell suspension with a density of 500 cells/mL was prepared and dispensed into six‐well plates, with each well containing 2 mL of culture medium. The cells were cultured for a duration of 2 weeks. After the incubation period, the cells were fixed and subjected to staining. Subsequently, the plates were washed, and images were captured for further analysis.

### 2.10 Transwell migration assay

Cell migratory capability was evaluated using an 8‐μm pore size transwell chamber (Costar). Transfected cells (2 × 104) were seeded in the upper chamber using serum‐free medium, while the lower chamber was filled with complete medium. After a 24‐h incubation period, cells that had migrated to the lower surface were fixed and stained with 4% paraformaldehyde and crystal violet. Subsequently, the cells were observed under an optical microscope (Olympus). The experiment was repeated three times to ensure reliable results.

### 2.11 M6A MeRIP qRT‐PCR

The Magna m6A methylated RNA immunoprecipitation (MeRIP) Kit from Millipore was utilized to quantify the levels of m6A‐modified FGD5‐AS1, following the provided instructions. Total RNA was extracted from Ishikawa and HHUA cells using Trizol reagent. The RNA was fragmented by heating at 94°C for 5 min and subjected to immunoprecipitation with 10 μg of anti‐m6A antibody in a 1 mL buffer containing an RNase inhibitor. Protein A/G magnetic beads were washed, added to the mixture, and incubated with rotation at 4°C for 2 h. The m6A‐modified RNA was eluted twice using 20 mM N6‐methyladenosine 5′‐monophosphate sodium salt at 4°C for 1 h, followed by purification using an RNA purification kit. The enrichment of m6A‐modified RNA was analysed through RT‐qPCR analysis.

### 2.12 Statistically analysis

Statistical analysis and data processing were carried out using SPSS software, version 22.0. The measurement data were presented as mean ± standard deviation (x ± s). Independent sample *t*‐tests were utilized for pairwise comparisons between groups. Differences among the various treatment groups were assessed using one‐way analysis of variance (one‐way anova). A *p*‐value below 0.05 was considered statistically significant.

## 3 RESULTS

### 3.1 FGD5‐AS1 is increased in chemoresistant EC cells

**FIGURE 1:**
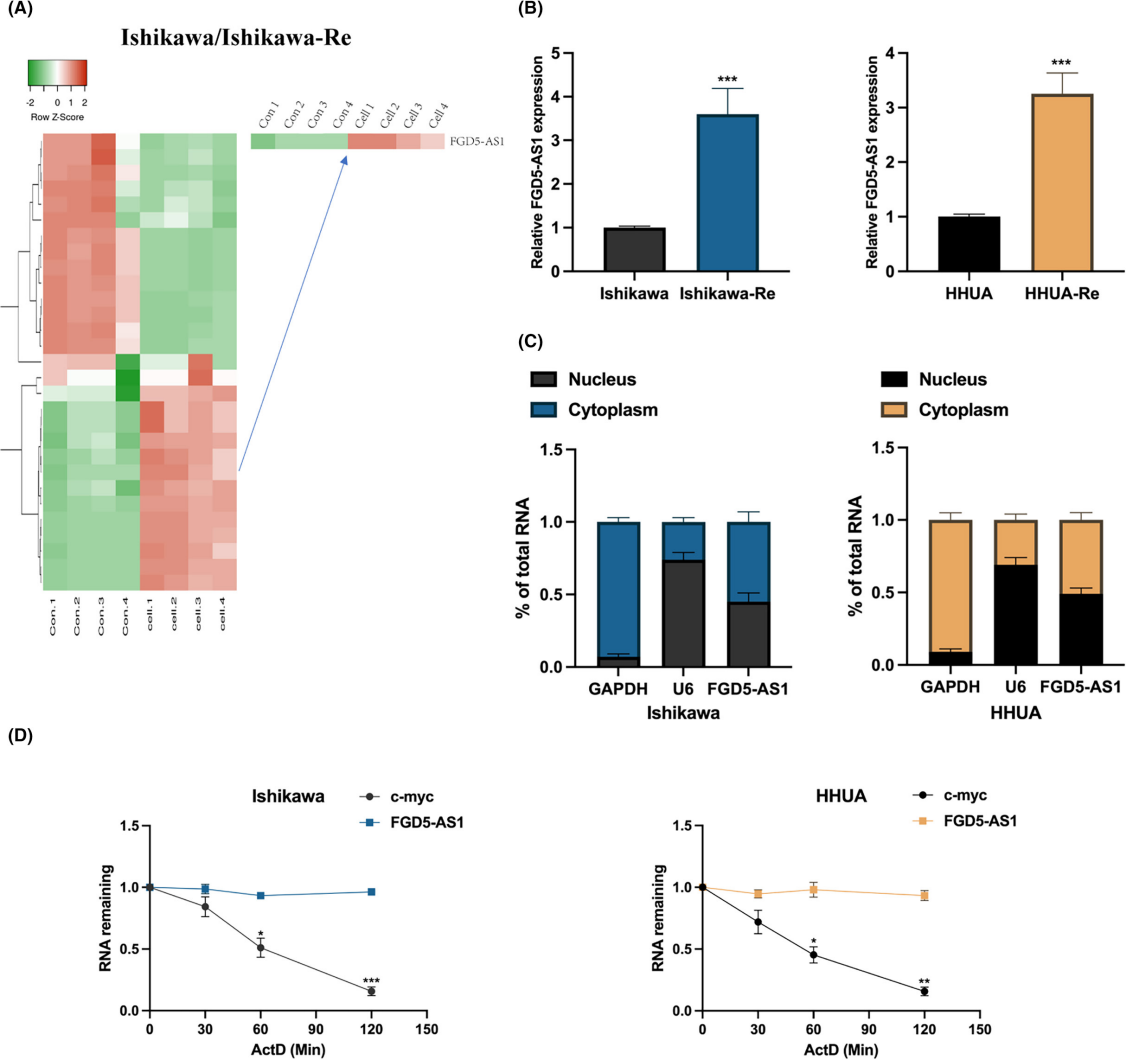
FGD5‐AS1 is increased in chemoresistant endometrial cancer (EC) cells. (A) Four pairs of PTX‐resistant Ishikawa and normal Ishikawa cells were subjected to long non‐coding RNA (LncRNA) microarray analysis, FGD5‐AS1 was found to be a most significantly upregulated LncRNA in PTX‐resistant Ishikawa cells. (B) FGD5‐AS1 expression in PTX‐resistant Ishikawa and HHUA cells were determined by qRT‐PCR. (C) Cellular distribution of FGD5‐AS1 in Ishikawa and HHUA cells were determined. (D) The stability of FGD5‐AS1 upon actinomycin D (ActD) treatment in a time‐dependent manner was determined by the qRT‐PCR assay. *p < 0.05, **p < 0.01, ***p < 0.001.

To explore the potential regulators involved in the resistance of EC cell to PTX treatment, we performed LncRNA microarray analysis on Ishikawa normal cell and PTX‐resistant Ishikawa cell. As shown in Figure 1A, FGD5‐AS1 was identified as a most significantly upregulated LncRNA in Ishikawa‐Resistant cells. The upregulation of FGD5‐AS1 were also be confirmed in PTX‐resistant Ishikawa and HHUA cells (Figure 1B). The localization analysis revealed that FGD5‐AS1 was predominantly located in the cytoplasm of Ishikawa and HHUA cells, suggesting that FGD5‐AS1 may play a significant role in cellular processes, as depicted in Figure 1C. In addition, the expression of FGD5‐AS1 remained stable even under treatment with actinomycin D (ActD), an inhibitor of RNA polymerase II, as shown in Figure 1D.

### 3.2 FGD5‐AS1 silencing re‐sensitive EC cells to PTX and attenuates immune escape

**FIGURE 2:**
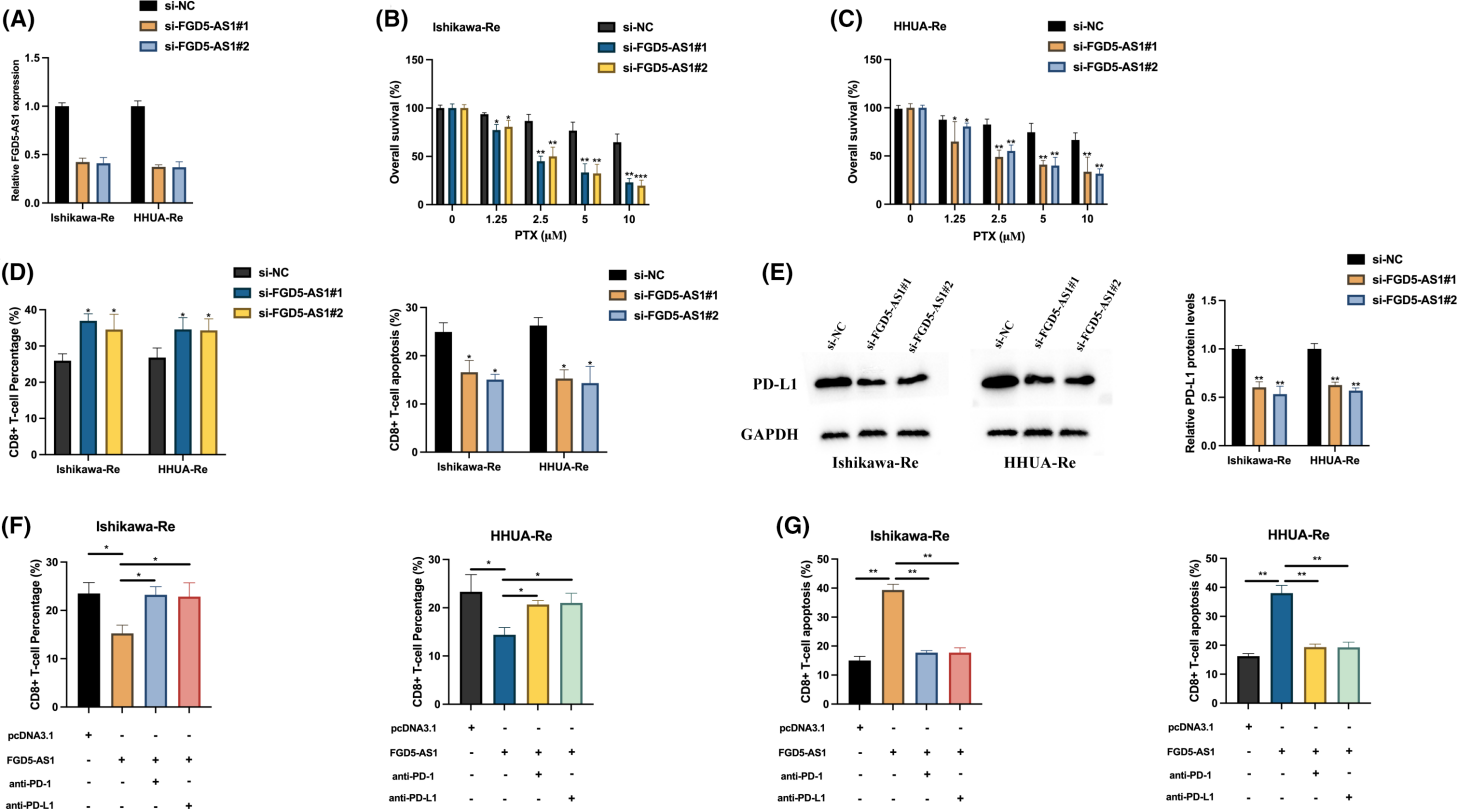
FGD5‐AS1 silencing re‐sensitive endometrial cancer (EC) cells to PTX and attenuates immune escape. (A) FGD5‐AS1 expression was measured by qRT‐PCR. (B, C) MTT assay was used to detect cell proliferation level, results were statically analysed. (D) The percentage of CD8^+^ T cells and the occurrence of apoptosis were measured in a co‐culture experiment involving CD8^+^ T cells and PTX‐resistant Ishikawa or HHUA cells transfected with si‐NC, si‐FGD5‐AS1#1 or si‐FGD5‐AS1#2. (E) The protein expression of PD‐L1 in PTX‐resistant Ishikawa or HHUA cell were detected by western blot. (F, G) In the co‐culture of CD8^+^ T cells and PTX‐resistant Ishikawa or HHUA cells transfected with si‐NC, si‐FGD5‐AS1#1 or si‐FGD5‐AS1#2, along with anti‐PD‐1 or PD‐L1 treatment, the CD8^+^ T‐cell percentage and apoptosis were examined. *p < 0.05, **p < 0.01, ***p < 0.001.

In order to investigate the functional role of FGD5‐AS1 in the development of PTX resistance, si‐NC, si‐FGD5‐AS1#1 and si‐FGD5‐AS1#2 were transfected into PTX‐resistant Ishikawa and HHUA cells to establish FGD5‐AS1 knockdown models. The downregulation of FGD5‐AS1 was confirmed through qRT‐PCR analysis, as shown in Figure 2A. The MTT assay results demonstrated that the knockdown of FGD5‐AS1 led to a significant resensitization of Ishikawa and HHUA cells to PTX treatment, as depicted in Figure 2B,C. Previous studies have reported the involvement of FGD5‐AS1 in immune escape by regulating the PD‐1/PD‐L1 checkpoint.^10^ Since the close relationship between chemoresistance and immune escape, therefore we examined if FGD5‐AS1 modulates the immune escape of PTX‐resistant Ishikawa and HHUA cells. The co‐culture of PTX‐resistant Ishikawa and HHUA cells with CD8^+^ T cells revealed that the knockdown of FGD5‐AS1 resulted in a significant increase in the ratio of CD8^+^ T cells and a decrease in the apoptosis rate of CD8^+^ T cells, as shown in Figure 2D. Additionally, the expression of PD‐L1 in PTX‐resistant Ishikawa and HHUA cells was decreased upon FGD5‐AS1 knockdown, as depicted in Figure 2E. Moreover, when co‐cultured with FGD5‐AS1 overexpressing PTX‐resistant Ishikawa and HHUA cells, the ratio of CD8^+^ T cells was increased and the apoptosis rate of CD8^+^ T cells was decreased. These effects were partially reversed by the addition of anti‐PD‐1 or anti‐PD‐L1 inhibitor, as demonstrated in Figure 2F,G. These findings suggest that FGD5‐AS1 regulates immune escape in PTX‐resistant Ishikawa and HHUA cells through modulation of the PD‐1/PD‐L1 checkpoint.

### 3.3 FGD5‐AS1 silencing inhibits EC cell proliferation and migration

**FIGURE 3:**
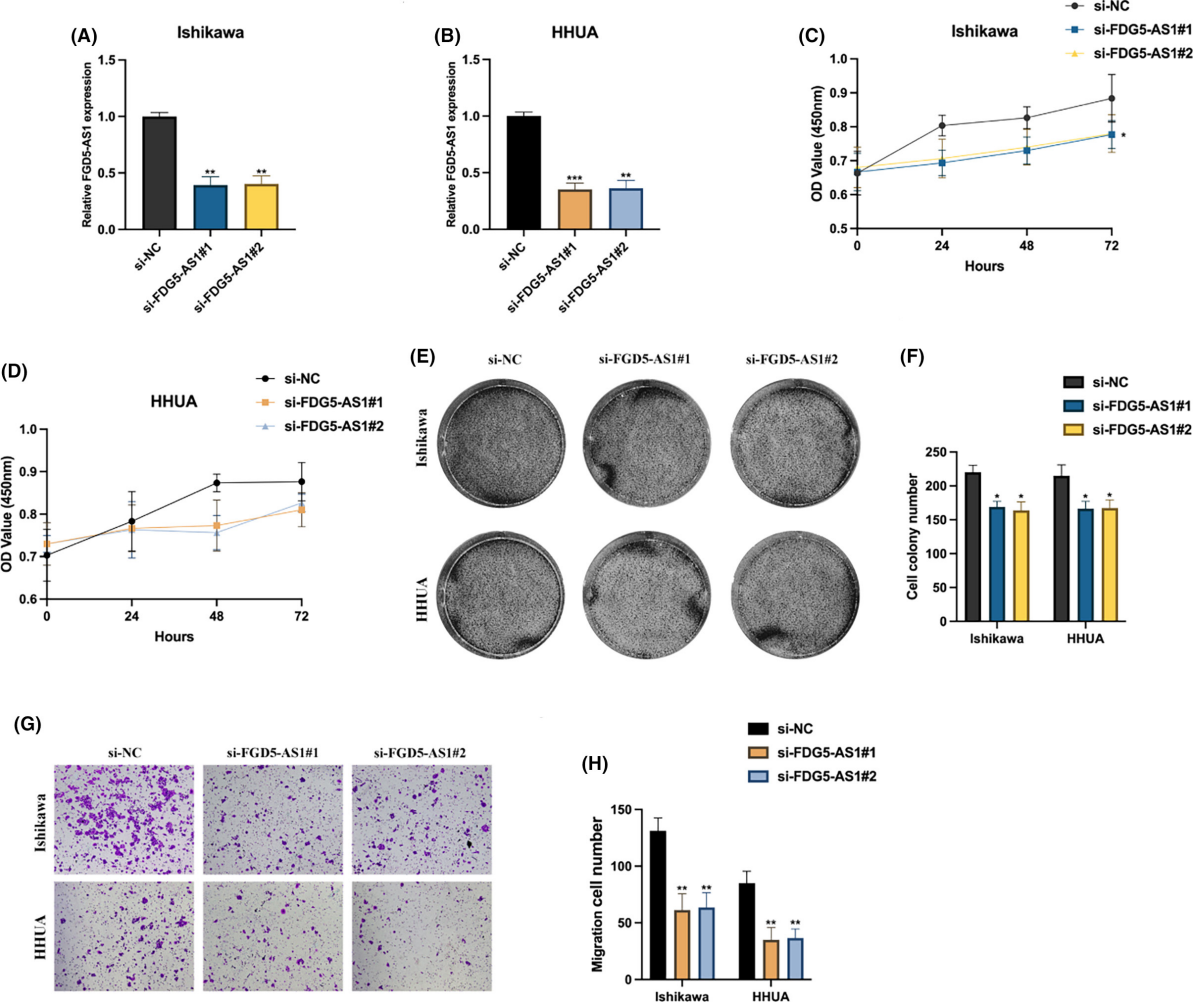
FGD5‐AS1 silencing inhibits endometrial cancer (EC) cell proliferation and migration. (A, B) si‐NC, si‐FGD5‐AS1#1 or si‐FGD5‐AS1#2 were introduced into Ishikawa or HHUA cells, results were measured by qRT‐PCR. (C, D) CCK‐8 assay was utilized to determine the proliferation level of FGD5‐AS1 knockdown cells. (E, F) Cell colony assay was performed to detect cell proliferation ability. (G, H) Transwell migration assay was used to evaluate cell migration levels. *p < 0.05, **p < 0.01, ***p < 0.001.

We next examined whether FGD5‐AS1 exerts function in EC tumorigenesis. FGD5‐AS1 knockdown cell models were generated using Ishikawa and HHUA cells (Figure 3A,B). CCK‐8 assay (Figure 3C,D) and cell colony assay (Figure 3E,F) results suggested that FGD5‐AS1 knockdown significantly reduced EC cell proliferation level. The transwell migration assay results indicated that FGD5‐AS1 knockdown also inhibited EC cell migration ability.

### 3.4 m6A methyltransferase METTL3 stabilizes FGD5‐AS1 expression

**FIGURE 4:**
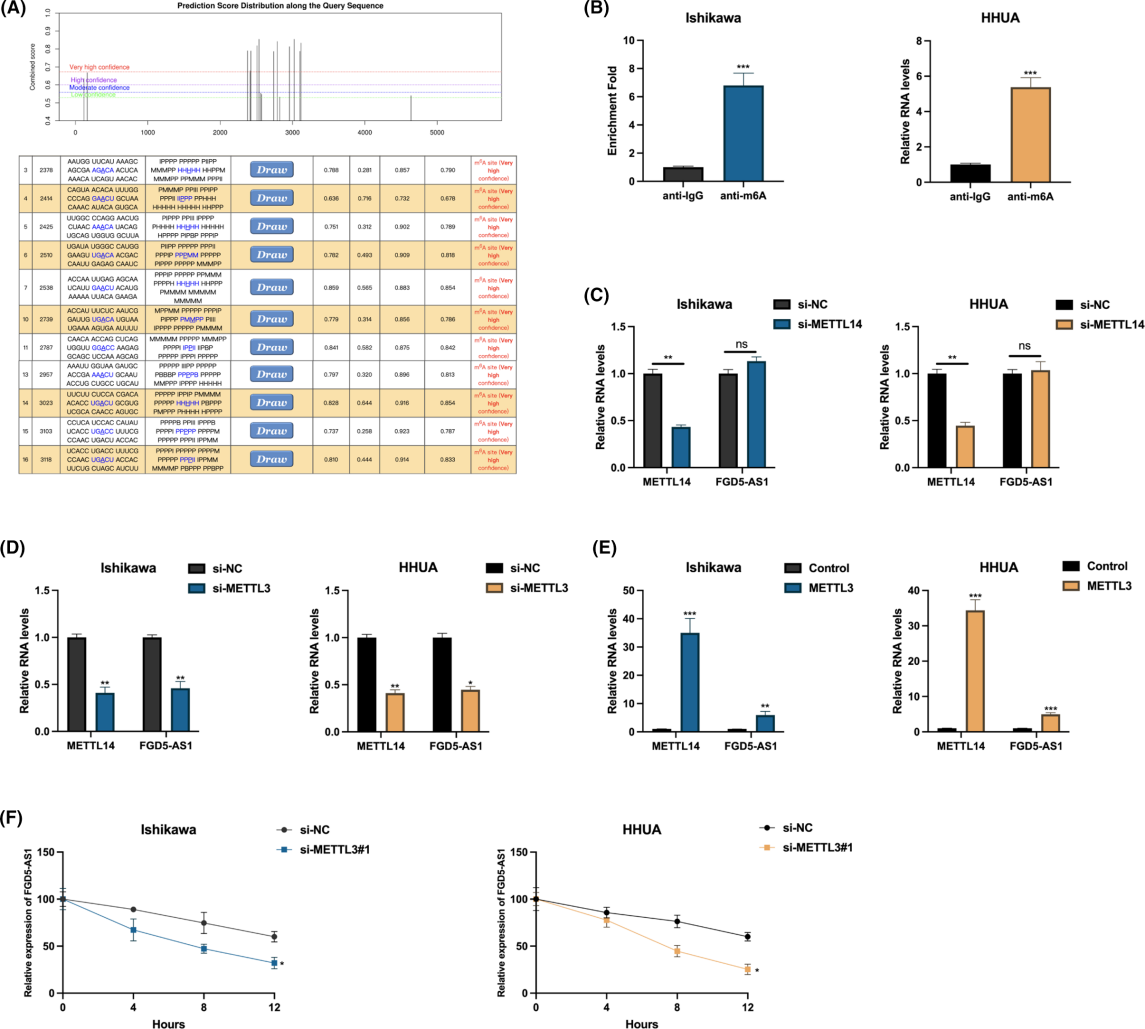
N6‐methyladenosine (m6A) methyltransferase METTL3 stabilizes FGD5‐AS1 expression. (A) A sequence‐based m6A modification site predictor (SRAMP: http://www.cuilab.cn/sramp/) was used to identify putative m6A modification site in FGD5‐AS1. (B) A methylated RNA immunoprecipitation (MeRIP) assay was utilized to show the m6A modification in FGD5‐AS1 in Ishikawa or HHUA cells. (C) METTL14 or FGD5‐AS1 expression in METTL14 knockdown cells were measured by qRT‐PCR. (D) METTL3 or FGD5‐AS1 expression in METTL3 knockdown cells were measured by qRT‐PCR. (E) METTL3 or FGD5‐AS1 expression in METTL3 overexpression cells were measured by qRT‐PCR. (F) FGD5‐AS1 in METTL3 knockdown cells pre‐treated with actinomycin D (ActD 5 μg/mL) was measured by qRT‐PCR at indicated time point. *p < 0.05, **p < 0.01, ***p < 0.001.

The prevailing internal RNA modification, widely observed in various transcripts, is referred to as m6A. This modification serves as a pivotal regulator of RNA degradation, stability and splicing processes.^11^, ^12^, ^13^ To explore the potential involvement of m6A in the post‐transcriptional regulation of FGD5‐AS1, we employed a sequence‐based predictor called SRAMP. This predictor was used to identify putative m6A modification sites within the nucleotide sequences of FGD5‐AS1. By utilizing SRAMP, we aimed to gain insights into the potential role of m6A in modulating the regulatory mechanisms of FGD5‐AS1 at the post‐transcriptional level,^14^ Multiple putative m6A modification sites were predicted for FGD5‐AS1, as depicted in Figure 4A. To validate the presence of m6A modifications in FGD5‐AS1, a MeRIP assay was conducted. The MeRIP assay results, presented in Figure 4B, indicated the presence of m6A modifications in FGD5‐AS1. To further explore which protein involves in the m6A modification of FGD5‐AS1, we first examined METTL14 and METTL3. It was found that METTL4 knockdown did not affect FGD5‐AS1 expression in Ishikawa and HHUA cells (Figure 4C). While knockdown or overexpression of METTL3 positively regulated FGD5‐AS1 expression (Figure 4D,E). Furthermore, the degradation of FGD5‐AS1 in Ishikawa and HHUA cells could be accelerated upon METTL3 knockdown (Figure 4F). Collectively, those results indicate that METTL3 is the upstream regulator of FGD5‐AS1 in EC cells.

### 3.5 METTL3 regulates the sensitivity of EC cells to PTX and immune escape via FGD5‐AS1

**FIGURE 5:**
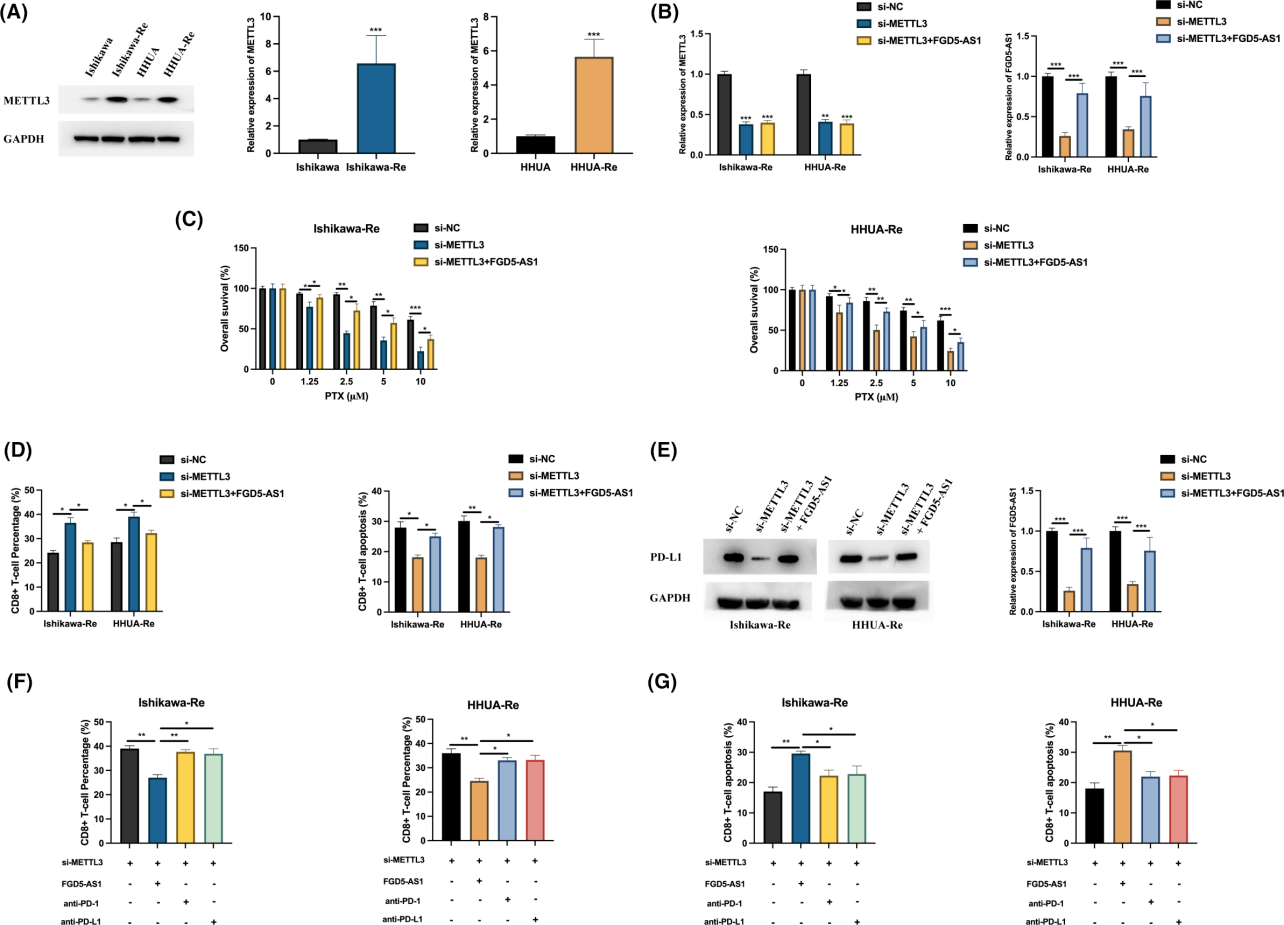
METTL3 regulates the sensitivity of endometrial cancer (EC) cells to PTX and immune escape via FGD5‐AS1. (A) Western blot analysis was performed to measure the expression of METTL3 in PTX‐resistant Ishikawa and HHUA cells. (B) PTX‐resistant Ishikawa and HHUA cells were transfected with si‐NC, si‐METTL3, si‐METTL3 and FGD5‐AS1, and the expression levels of METTL3 and FGD5‐AS1 were determined using qRT‐PCR. (C) The proliferation level of PTX‐resistant cells was assessed using the MTT assay, and the results were subjected to statistical analysis. (D) In a co‐culture experiment involving CD8^+^ T cells and PTX‐resistant Ishikawa or HHUA cells transfected with si‐NC, si‐METTL3, si‐METTL3 and FGD5‐AS1, the percentage of CD8^+^ T cells and the occurrence of apoptosis were measured. (E) Western blot analysis was performed to examine the protein expression of PD‐L1 in PTX‐resistant Ishikawa or HHUA cells transfected with si‐NC, si‐METTL3, si‐METTL3 and FGD5‐AS1. (F, G) In the co‐culture of CD8^+^ T cells and PTX‐resistant Ishikawa or HHUA cells transfected with si‐NC, si‐METTL3, si‐METTL3 and FGD5‐AS1, in combination with anti‐PD‐1 or PD‐L1 treatment, the percentage of CD8^+^ T cells and the occurrence of apoptosis were evaluated. Statistical significance was denoted by *p < 0.05, **p < 0.01, and ***p < 0.001.

METTL3 expression in PTX‐resistant Ishikawa and HHUA cells were upregulated compared with normal cells (Figure 5A). To investigate the biological relationship between METTL3 and FGD5‐AS1 in EC, first we transfected si‐NC, si‐METTL3 and si‐METTL3 with FGD5‐AS1 into PTX‐resistant Ishikawa and HHUA cells (Figure 5B). It was found that METTL3 knockdown significantly inhibited PTX‐resistant Ishikawa and HHUA cell proliferation, but this effect could be blocked by FGD5‐AS1 overexpression (Figure 5C). Following the co‐culture of PTX‐resistant Ishikawa and HHUA cells with CD8^+^ T cells, the knockdown of METTL3 resulted in an increased ratio of CD8^+^ T cells and a decreased rate of CD8^+^ T‐cell apoptosis. Importantly, this effect was reversed when FGD5‐AS1 was present, as illustrated in Figure 5D. These findings suggest that METTL3, in conjunction with FGD5‐AS1, modulates the ratio of CD8^+^ T cells and the apoptosis rate. The PD‐L1 expression in PTX‐resistant Ishikawa and HHUA cell, downregulated by METTL3 knockdown, could be upregulated by FGD5‐AS1 (Figure 5E). Moreover, anti‐PD‐l or anti‐PD‐L1 inhibitor blocked the FGD5‐AS1 effect on CD8^+^ T cell, which co‐cultured with METTL3 knockdown PTX‐resistant Ishikawa and HHUA cell (Figure 5F,G). Those results suggest that METTL3 enhances the resistance EC cells to PTX treatment and blocked the immune escape to PTX‐resistant EC cells via PD‐1/PD‐L1 checkpoint through regulating FGD5‐AS1 expression.

### 3.6 METTL3 modulates EC cell proliferation and migration through FGD5‐AS1

**FIGURE 6:**
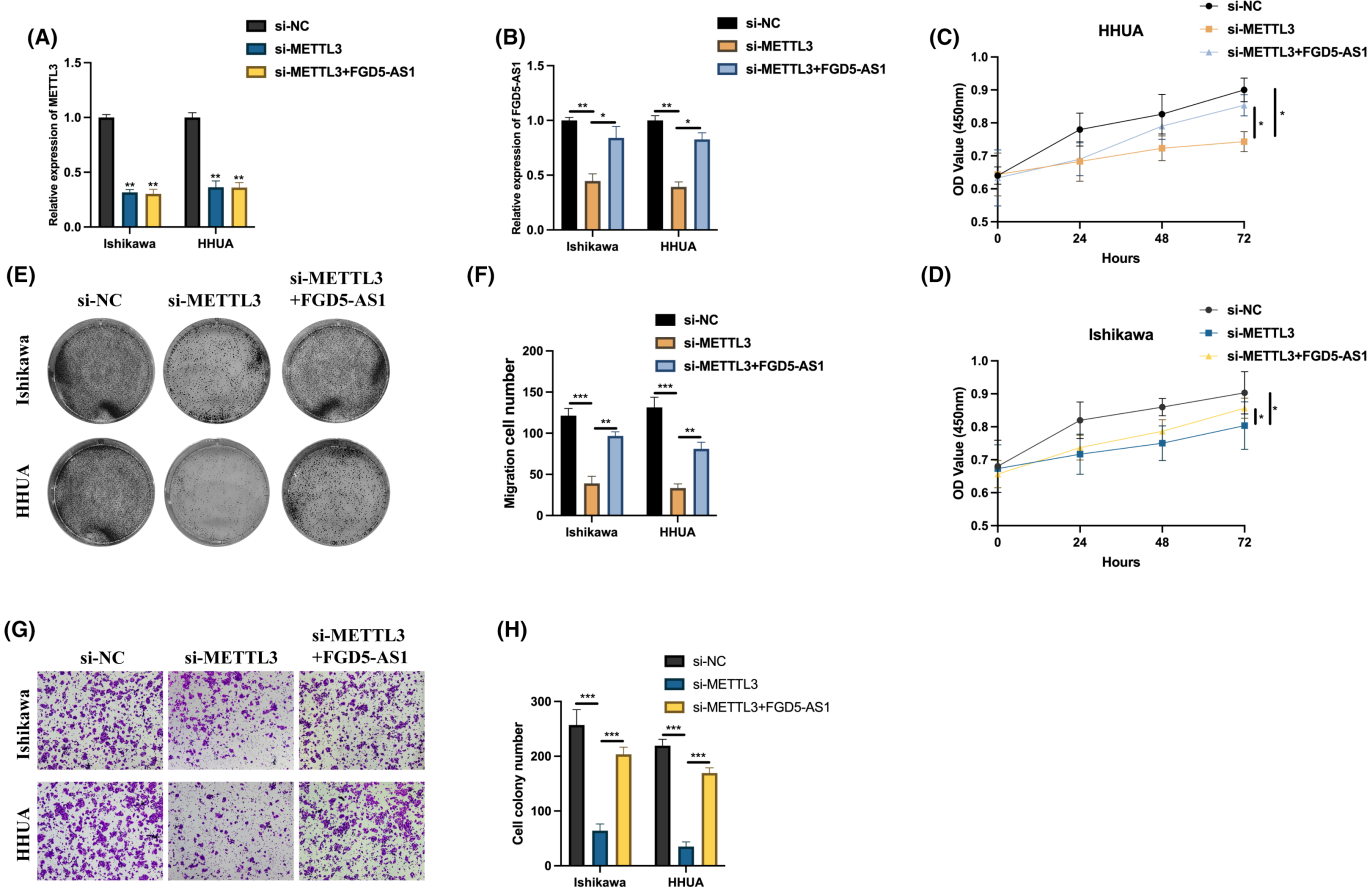
METTL3 modulates endometrial cancer (EC) cell proliferation and migration through FGD5‐AS1. (A, B) METTL3 and FGD5‐AS1 expression in Ishikawa or HHUA cells introduced with si‐NC, si‐METTL3, si‐METTL3 and FGD5‐AS1 were measured by qRT‐PCR. (C, D) CCK‐8 assay was utilized to determine the proliferation level of each cell. (E, F) Cell colony assay was performed to detect cell proliferation ability. (G, H) Transwell migration assay was used to evaluate cell migration levels. *p < 0.05, **p < 0.01, ***p < 0.001.

To identify the role of METTL3/FGD5‐AS1 axis in EC tumorigenesis, si‐NC, si‐METTL3 and si‐METTL3 with FGD5‐AS1 were introduced into Ishikawa and HHUA cells (Figure 6A). The proliferation level of EC cells examined by the CCK‐8 assay (Figure 6C,D) and cell colony assay (Figure 6E,F) were inhibited by METTL3 knockdown, and this effect was inhibited by FGD5‐AS1. The same phenomena were also observed in the transwell migration assay (Figure 6G,H).

## 4 DISCUSSION

EC is a malignancy that originates in the endometrium, which is the inner lining of the uterus. It is a type of cancer that affects primarily women and can lead to various symptoms and complications.^3^, ^15^ Patients with EC commonly present with symptoms such as abnormal vaginal bleeding, which may include postmenopausal bleeding or bleeding between menstrual periods. Other symptoms may include vaginal discharge, pelvic pain or discomfort, and changes in urinary or bowel habits. These symptoms should be evaluated by a healthcare professional, as they can be indicative of EC or other gynaecological conditions. Furthermore, if left untreated, EC can lead to the complications such as metastasis (spread of cancer to other parts of the body), obstruction of the urinary or digestive tract, and adverse effects on overall health and well‐being. Therefore, it is important for individuals experiencing these symptoms to seek medical attention for proper diagnosis and management.^16^, ^17^ The exact cause of EC is still not fully understood, but long‐term exposure to oestrogen, endometrial hyperplasia and a family history of the disease are recognized as important risk factors for EC.^18^ Chemotherapy is a standard treatment for EC and is aimed at destroying cancer cells or slowing down their growth. However, the development of chemoresistance, where cancer cells become resistant to the effects of chemotherapy drugs, can pose a significant challenge in the management of EC.^19^ Indeed, understanding the molecular mechanisms underlying chemoresistance in EC is essential for developing strategies to enhance chemosensitivity and improve treatment outcomes. Identification of the functional LncRNA FGD5‐AS1 in PTX‐resistant EC cells is a significant finding. The study highlights that FGD5‐AS1 contributes to the resistance of EC cells to PTX treatment and affects the immune escape of PTX‐resistant EC cells through the PD‐1/PD‐L1 checkpoint. These findings emphasize the important role of LncRNAs in the development of chemoresistance in EC cells and shed light on a promising research direction for investigating PTX resistance in EC. By uncovering the specific mechanisms by which FGD5‐AS1 influences chemoresistance and immune escape, potential therapeutic targets and strategies can be identified to enhance the effectiveness of PTX treatment and improve patient outcomes.

The co‐inhibitory PD‐1 pathway has garnered significant attention in cancer research due to its crucial role in regulating the immune checkpoint response of cytotoxic T cells. Tumour cells exploit this pathway to evade immune surveillance and establish immune tolerance. The interaction between PD‐1 expressed on T cells and its ligand PD‐L1 expressed on tumour cells results in T‐cell exhaustion and impaired anti‐tumour immune responses.^20^ Despite the demonstrated effectiveness of checkpoint blockade immunotherapy in certain cancer types, including oesophageal cancer (EC), the response rates among patients can vary significantly, with only a small proportion of individuals showing positive responses to the treatment within a larger cohort. This variability in patient response suggests the presence of additional factors that influence therapeutic outcomes. Several mechanisms have been proposed to contribute to resistance to PD‐1 pathway blockade, such as the upregulation of alternative immune checkpoints, genetic alterations, characteristics of the tumour microenvironment and patterns of immune cell infiltration. Further investigation is required to unravel the complexities underlying the differential response to immunotherapy and to identify potential biomarkers that can predict treatment outcomes and guide personalized therapeutic approaches not only in EC but also in other types of cancer.^21^, ^22^ Previous studies have indicated the involvement of FGD5‐AS1 in immune evasion by modulating the PD‐1/PD‐L1 checkpoint. However, it remains unclear whether FGD5‐AS1 plays a similar role in PTX‐resistant EC. Our findings demonstrate that blocking FGD5‐AS1 activity leads to a reduction in immune evasion, as evidenced by changes in CD8^+^ T‐cell percentage and apoptosis rates observed after co‐culture with PTX‐resistant EC cells. Furthermore, our results indicate that FGD5‐AS1 also regulates immune evasion through the PD‐1/PD‐L1 checkpoint in PTX‐resistant EC. These findings shed light on the potential role of FGD5‐AS1 in modulating immune responses and highlight its relevance in PTX‐resistant EC.

m6A modifications are the most common internal modifications observed in eukaryotic RNA. These modifications have been implicated in the regulation of diverse RNA molecules, playing crucial roles in various cellular processes.^23^ The m6A modification is a dynamic and reversible process regulated primarily by the three classes of enzymes involved in methylation. The enzymes responsible for the formation of m6A include METTL3, METTL14, Wilms' tumour 1‐associated protein (WTAP), RNA‐binding motif protein 15 (RBM15), methyltransferase‐like protein 16 (METTL16) and vir‐like m6A methyltransferase‐associated protein (VIRMA). These enzymes play key roles in the addition of methyl groups to adenosine residues, thereby influencing RNA structure, stability and various biological processes.^24^ Among the enzymes involved in m6A methylation, METTL3, METTL14 and WTAP form a complex called the m6A writer complex. This complex plays a central role in catalysing the addition of m6A modifications and is crucial for the proper functioning of m6A methylation. The writer complex ensures precise and regulated m6A modification on target RNA molecules, thereby influencing their processing, stability, localization and function. It acts as a key regulatory module in the m6A modification pathway and is essential for the dynamic regulation of RNA metabolism and gene expression.^25^ The m6A modifications introduced by the writer complex can undergo dynamic reversal through the action of two main types of demethylases. These demethylases include obesity‐associated protein (FTO) and alpha‐ketoglutarate‐dependent dioxygenase homologue 5 (ALKBH5). FTO and ALKBH5 enzymes could recognize and remove the methyl groups from m6A‐modified RNA, thereby reversing the m6A modification. The demethylation process mediated by FTO and ALKBH5 provides a means of fine‐tuning and regulating the m6A landscape, allowing for dynamic changes in RNA structure and function.^26^ The enzymes involved in m6A modification and the m6A modification process play crucial roles in various biological processes, including RNA stability, splicing, transport, translation and decay. Dysregulation of m6A modification has been implicated in numerous diseases, such as cancer, neurological disorders and metabolic disorders. Understanding the mechanisms and functional consequences of m6A modifications is an active area of research with the potential to uncover new insights into gene regulation and develop novel therapeutic strategies. In the context of investigating the molecular mechanisms of FGD5‐AS1 in PTX‐resistant EC cells, the upstream regulator of FGD5‐AS1 was examined. The findings revealed that METTL3, rather than METTL14, modulates the m6A modification of FGD5‐AS1 and upregulates its expression. Additionally, METTL3 was found to be a functional gene in PTX‐resistant EC cells. METTL3 enhanced PTX resistance and inhibited immune escape in EC cells, and these biological functions were exerted through its regulation of FGD5‐AS1 expression. These findings highlight the role of METTL3 and FGD5‐AS1 in PTX resistance and immune modulation in EC cells, providing valuable insights into potential therapeutic targets and strategies for overcoming drug resistance and immune evasion in EC.

Our study has provided partial insights into the molecular mechanisms and cellular behaviours governed by FGD5‐AS1 in PTX‐resistant EC cells. However, several avenues of research remain unexplored. These include investigating the clinical significance of FGD5‐AS1 and conducting in vivo validation studies to further elucidate its role in EC. Collectively, our results identified a functional LncRNA FGD5‐AS1 in PTX‐resistant EC cells and elucidated that METTL3 could regulate the m6A modification in FGD5‐AS1. The biological functions of METTL3/FGD5‐AS1 axis have been partially revealed. Our results may provide new direction of PTX‐resistant EC fundamental research and therapeutic target development.

## AUTHOR CONTRIBUTIONS

**Min Hao:** Data curation (equal); formal analysis (equal); software (equal); validation (equal). **Tianjie Li:** Data curation (equal); investigation (equal); validation (equal); visualization (equal). **Ling Xiao:** Data curation (equal); validation (equal). **Yun Liu:** Conceptualization (equal); project administration (equal); supervision (equal); validation (equal); writing – original draft (equal).

## CONFLICT OF INTEREST STATEMENT

The authors declare that they have no competing interests.

## Data Availability

The data from this research can be obtained from corresponding author upon reasonable request.
